# Fast-track endovascular aneurysm repair: rationale and design of the multicenter Least Invasive Fast-Track EVAR (LIFE) registry

**DOI:** 10.1186/s12872-015-0167-1

**Published:** 2015-12-19

**Authors:** Zvonimir Krajcer, Venkatesh Ramaiah, Meredith Huetter

**Affiliations:** St. Luke’s Episcopal Hospital, Houston, TX USA; Arizona Heart Institute, Phoenix, AZ USA; TriVascular, Inc., Santa Rosa, CA USA

**Keywords:** Abdominal aortic aneurysm, Endovascular, Fast-track, Ovation, Protocol, Stent graft

## Abstract

**Background:**

Considerable technological advancements have recently been made with endovascular stent grafts for the treatment of abdominal aortic aneurysm (AAA). However, there is opportunity to further improve the efficiency of endovascular aneurysm repair (EVAR), which may yield better patient outcomes and lower perioperative treatment costs.

**Methods/Design:**

The Least Invasive Fast-Track EVAR (LIFE) registry was developed to determine the clinical utility and cost effectiveness of the Ovation® Prime stent graft when used under least invasive conditions using a defined fast-track protocol. The LIFE study is a prospective multicenter post-market registry of the ultra-low profile (14F) Ovation Prime stent graft when used in the treatment of patients with AAA using a fast-track protocol, consisting of appropriate patient selection, bilateral percutaneous access, avoidance of general anesthesia and intensive care unit admission, and next-day discharge. The primary endpoint of the study is the proportion of subjects that experience a major adverse event within 30 days of the initial procedure. Primary endpoint data will be compared to a target performance goal. A total of 250 subjects will be enrolled at up to 40 sites in the United States. The first subject in this study was enrolled in October 2014 and enrollment is anticipated to continue through mid-2016.

**Discussion:**

The recent development of ultra low-profile stent grafts enables EVAR using least invasive methods. A structured fast-track EVAR protocol may yield clinical and cost benefits versus standard EVAR.

**Trial registration:**

ClinicalTrials.gov Identifier: NCT02224794

## Background

Over the last decade, considerable advancements have been made with endovascular stent grafts for the treatment of abdominal aortic aneurysm (AAA). Early studies with endovascular aortic repair (EVAR) demonstrated significantly lower perioperative morbidity and mortality rates compared to open surgical resection [1]. Consequently, EVAR has rapidly become the standard of care for AAA repair at most centers. Studies with latest-generation stent grafts have focused on improving treatment durability and expanding EVAR eligibility to accommodate patients with challenging aortoiliac anatomy. Despite this recent progress, considerable opportunity exists to further improve the efficiency of EVAR, which may yield even better patient outcomes and lower perioperative treatment costs.

Typical EVAR cases involve vascular access via surgical exposure of the common femoral artery, general anesthesia, a 24-h intensive care unit (ICU) stay, and a 3-day hospitalization [2], all of which increase morbidity and significantly contribute to the total cost of EVAR. In the current economic climate, there is heightened scrutiny on health care resource utilization such that opportunities for improved surgical- and hospital-related efficiency must continue to be explored. Fast-track hospital care pathways have been adopted with increasing frequency for open aortic surgery, resulting in shorter ICU [3, 4] and hospital [5, 6] stays with reductions in morbidity [3, 4, 6] compared to traditional surgical practices. However, experience with fast-track EVAR remains limited. The common premise of fast-track EVAR includes well-selected patients with low-risk of periprocedural complications, bilateral percutaneous vascular access, and regional anesthesia with or without conscious sedation, all of which enable faster patient recovery and expedite hospital discharge. The largest study of fast-track EVAR involved 915 patients treated with bilateral percutaneous access and local anesthesia/conscious sedation [7]. Treatment success was achieved in 94 % of cases, mean hospital stay was 1.3 days, and 30-day mortality was only 0.6 %. The clinical and cost benefits associated with fast-track EVAR are potentially substantial, but currently are not well characterized. The Least Invasive Fast-Track EVAR (LIFE) registry was developed to explore the clinical utility and cost effectiveness of a defined fast-track EVAR protocol in patients undergoing elective AAA repair.

## Methods/Design

The LIFE registry is a prospective, nonrandomized, multicenter post-market study that will enroll 250 patients at up to 40 sites in the United States. The protocol for this clinical trial has been approved by the institutional review board (IRB) at each participating site. Recruitment is ongoing and additional sites, up to 40 total sites, will be added to those already named in Table 1. All patients provided informed consent before study participation. Patient enrollment began in October 2014. This study was prospectively registered at www.clinicaltrials.gov (NCT02224794).

**Table 1:** Study Sites and Institutional Review Board

Site Name	Institutional Review Board
Central Arkansas Veterans Healthcare System	Central Arkansas Veterans Healthcare System IRB
NCH Healthcare Systems	NCH IRB
Temple University Hospital	WIRB
Wellmont Holston Valley MC	Wellmont Health System IRB
Morton Plant Hospital	BayCare Health System IRB
Swedish Heart and Vascular	WIRB
Bend Memorial Clinic	WIRB
Hartford Hospital	Hartford Health Care IRB
WVU Hospital	West Virginia University IRB
St. Luke’s Episcopal Hospital	BRANY IRB
Palomar Medical Center	WIRB
SIH Memorial Hospital	SIH IRB
Sutter Memorial Hospital	WIRB
Chandler Regional Medical Center	East Valley Regional IRB
Syracuse VA Medical Center	Syracuse VAMC IRB
Southern Ohio Medical Center	Southern Ohio Medical Center IRB
Jersey Shore University Medical Center	WIRB
St. Joseph Mercy Oakland	St. Joseph Mercy IRB
Phoenix St. Luke‘s Medical Center	WIRB
Medical University of South Carolina	MUSC IRB III
OhioHealth Research Institute	WIRB
Arizona Heart Institute	WIRB
Heart Hospital of New Mexico	WIRB
Tuscon Medical Center	WIRB
Harrison Medical Center	WIRB and Harrison Medical Center IRB
Bakersfield Heart Hospital	WIRB
Saint Jospeh Hospital	WIRB
Lutheran Medical Center	Lutheran Medical Center IRB
Middlesex Hospital	Middlesex Hospital IRB
Sacred Heart Hospital	Sacred Heart Clinical IRB
Memorial Hospital of Jacksonville (FCCI)	WIRB
Gwinnett Medical Center	WIRB
Scottsdale Healthcare-Osborn	WIRB
Northern Michigan Regional Hospital	WIRB

### Participants

Eligible patients are adults with AAA requiring elective intervention who have anatomy suitable for endovascular repair. Complete inclusion and exclusion criteria are reported in Table 2. Pre-treatment assessments will include medical and surgical history, laboratory tests for creatinine and pregnancy, spiral contrast-enhanced computed tomography, and questionnaires related to groin pain severity and health-related quality of life. Patients will be enrolled and treated with EVAR if the investigator determines that bilateral percutaneous access, avoidance of general anesthesia and ICU stay, and next-day hospital discharge are feasible based on medical history and aortoiliac anatomy. Following enrollment, patients will remain in the study regardless of whether all components of the fast-track program are completed. Patient outcomes will be recorded through hospital discharge and at the 30-day follow-up visit per the schedule of activities in Table 3.

**Table 2:** Main study entry criteria

Main inclusion criteria
− Age ≥ 18 years
− Male or non-pregnant female
− Candidate for elective open surgical AAA repair
− AAA >5.0 cm diameter, increased ≥ 0.5 cm diameter in last 6 months, or maximum diameter > 1.5x adjacent non-aneurysmal aorta
− Suitable anatomy for endovascular repair with the Ovation Prime Stent Graft
− Suitable anatomy to allow Perclose ProGlide Suture-Mediated Closure System via the pre-close technique
Main exclusion criteria
− Dissecting or acutely ruptured AAA
− Acute vascular injury
− Prior AAA or iliac artery repair
− Mycotic AAA or active systemic infection
− Unstable angina
− Unstable peripheral artery disease with critical limb ischemia
− Congestive heart failure
− Myocardial infarction or stroke within the past 3 months
− Need for renal artery coverage (e.g. Chimney graft)
− Planned adjunctive devices (e.g. renal stent)
− Major surgery or interventional procedure within the past 30 days
− Connective tissue disease (e.g. Marfan’s or Ehler’s–Danlos syndrome)
− History of bleeding disorder or refuses blood transfusions
− Dialysis-dependent renal failure or serum creatinine >2.0 mg/dl
− Morbid obesity (BMI ≥40 kg/m^2^)
− Home oxygen use
− Patient admitted from skilled nursing facility
− Life expectancy < 1 year
− Anticipated inability to discharge patient within 1 day
− Participation in investigational device or drug clinical trial
− Intolerance/hypersensitivity to anticoagulation, contrast media, or stent graft components

**Table 3:** Schedule of study activities

Procedure	Baseline	Procedure	Post-Procedure	1 Month
Medical/surgical history	X			
Spiral contrast-enhanced CT	X			X
Laboratory assessment (creatinine, serum pregnancy)	X			
Quality of life (EQ-5D)	X			X
Groin pain (Wong-Baker FACES Pain Rating Scale)	X		X	X
Endovascular AAA repair		X		
Adverse events		X	X	X
Assessment of ambulation and normal diet			X	

### Device

The endograft under study is ideally suited for use in a fast-track EVAR program. The Ovation Prime stent graft is characterized by a tri-modular design with the aortic body delivered via a flexible hydrophilic-coated 14 Fr OD catheter, the smallest profile of any currently commercially available stent grafts. The aortic body is comprised of a low permeability PTFE graft and a suprarenal nitinol stent with integral anchors to achieve active fixation to the aortic wall. The aortic body contains a network of inflatable channels and sealing rings that are filled during deployment with a low viscosity, radiopaque fill polymer that cures in situ to create a conformable seal to the aortic neck. The Ovation Prime iliac limbs are comprised of highly flexible nitinol stents encapsulated in low-permeability PTFE that are packaged in ultra-low profile 13-14F OD delivery system.

### Outcomes

The primary endpoint of the LIFE registry is the incidence of major adverse events through 30 days follow-up. Major adverse events are defined as any of the following events: death, myocardial infarction, stroke, renal failure, respiratory failure, paralysis, bowel ischemia, or procedural blood loss ≥ 1000 cc. All adverse events will be adjudicated by a Clinical Events Committee (CEC). Secondary endpoints of the LIFE registry include operative details, technical success, procedure- and device-related complications, patient convalescence, and ability to successfully complete all components of the fast-track protocol (Table 4). Patients will be followed in the LIFE registry for 30 days post-treatment and will undergo lifelong surveillance thereafter. A 30-day primary endpoint is appropriate since the benefits of a fast-track EVAR program are anticipated to be realized entirely in the perioperative period.

**Table 4:** Secondary endpoints

− Serious and non-serious adverse events
− Access technical success (i.e. procedure successfully completed with bilateral percutaneous access)
− Treatment success (i.e. successful completion of least invasive protocol through discharge)
− Blood loss, including if transfusion required
− Percent of procedures completed without general anesthesia
− Anesthesia time
− Procedure time
− Contrast volume
− Fluoroscopy time
− Time to hemostasis
− Time to ambulation
− Time to normal diet
− Groin pain
− Quality of life
− Percent of subjects discharged without ICU admission
− Length of ICU stay, if required
− Length of hospital stay
− Percent of subjects discharged within only one overnight stay
− Freedom from type I & III endoleak
− Freedom from AAA rupture
− Freedom from conversion to open repair
− Freedom from AAA-related secondary interventions
− Freedom from mortality

### Hypothesis

The primary hypothesis of the LIFE registry is that the incidence of major adverse events through 30 days will be less than a target performance goal (TPG) of 10.4 %. The TPG was determined by using the 95 % upper-bound confidence interval of the 30-day major adverse event rate in the Ovation® pivotal trial (5.4 %) [8] and adding a 5 % margin.

### Statistical methods

The sample size for the LIFE registry was determined using the exact binomial method based on a one-sided 5 % significance level, an anticipated 5 % major adverse event rate, and no more than 20 % attrition. Based on these assumptions, a sample size of 250 subjects provides at least 87 % power to test the primary hypothesis. Intent-to-treat analyses will be performed, which include all patients enrolled in the registry regardless of whether the fast-track protocol is completed successfully. Subjects will be considered enrolled when the delivery catheter is inserted into the vasculature. Continuous data will be reported using mean and standard deviation or median, minimum, and maximum, depending on normality assumptions. Categorical data will be reported with counts and percentages. The incidence of major adverse events will be compared to the TPG by calculating a one-sided 95 % Wilson upper confidence interval limit. Subgroup analyses will be performed for: a) patients who complete the fast-track EVAR protocol, b) patients who undergo bilateral percutaneous access but do not complete the fast-track EVAR protocol, and c) patients who do not undergo bilateral percutaneous access.

### Study progress

The first patient in this study was enrolled in October 2014 and enrollment is anticipated to continue through mid-2016. As of October 4, 2015, 149 patients have been enrolled at over 25 sites in the United States.

## Discussion

The recent development of ultra low-profile stent grafts enables EVAR using least invasive methods. However, strict patient selection criteria are crucial to improving outcomes of a fast-track EVAR program. Ideal candidates have access vessels free of heavy calcification or extreme tortuosity and have no major risk factors for prolonged procedure or hospitalization such as ruptured AAA, renal failure, and severe comorbid conditions. Bilateral percutaneous vascular access results in higher technical success, less blood loss, fewer complications, and shorter hospital stay compared to surgical cutdown [7, 9–15]. Avoidance of general anesthesia is associated with lower rates of mortality and morbidity and shorter intensive care unit and hospital stays compared to regional anesthesia [16]. Next-day hospital discharge with no ICU stay has obvious cost benefits, provided that patient safety is not compromised. The observed advantages of each separate component may potentially yield synergistic clinical and cost benefits when utilized collectively in a structured fast-track EVAR protocol. The results of the LIFE study with the Ovation Prime stent graft are anticipated to be available in late 2016.
